# Lonely, stressed-out moms: Does the postindustrial social experience put women at risk for perinatal mood disorders?

**DOI:** 10.1093/emph/eoae025

**Published:** 2024-10-04

**Authors:** Elena Bridgers, Molly M Fox

**Affiliations:** Department of Anthropology, University of California, Los Angeles, CA, 90095, USA; Department of Anthropology, University of California, Los Angeles, CA, 90095, USA; Department of Psychiatry & Biobehavioral Sciences, Semel Institute for Neuroscience and Human Behavior, David Geffen School of Medicine, University of California, Los Angeles, CA 90095, USA

**Keywords:** allomothering, social support, perinatal mental health, perinatal depression, perinatal mood and anxiety disorders, evolutionary mismatch

## Abstract

Perinatal mood and anxiety disorders (PMADs) are estimated to affect as many as 17.7% of mothers in agricultural and postindustrial societies. Various lines of research converge to suggest that PMADs may be ‘diseases of modernity’, arising from a mismatch between the environments in which humans evolved over hundreds of thousands of years and contemporary postindustrial lifestyles. Here we highlight the social context of childrearing by focusing on three sources of mismatch associated with PMADs: closer interbirth spacing, lack of allomaternal support and lack of prior childcare experience. The transitions to agriculture and industrialization disrupted traditional maternal support networks, allowing closer birth spacing without compromising infant survival but increasing maternal isolation. Caring for closely spaced offspring is associated with high levels of parenting stress, and poses a particular challenge in the context of social isolation. The mother’s kin and community play a critical role in allomothering (childcare participation) in all contemporary hunter-gatherer societies, facilitating a system of simultaneous care for children of a range of ages with unique age-specific needs. The absence of social support and assistance from allomothers in postindustrial societies leaves mothers at increased risk for PMADs due to elevated caregiving burdens. Furthermore, the traditional system of allomothering that typified human evolutionary history afforded girls and women experience and training before motherhood, which likely increased their self-efficacy. We argue that the typical postindustrial motherhood social experience is an evolutionary anomaly, leading to higher rates of PMADs.

## MATERNAL MENTAL HEALTH IN POSTINDUSTRIAL SOCIETIES

Perinatal mood and anxiety disorders (PMADs) are estimated to affect as many as 17.7% of women worldwide [1]. PMADs can be classified as any kind of mood or anxiety disorder appearing in mothers from conception to within the first year after birth and may include depression, anxiety, obsessive-compulsive disorder, posttraumatic stress disorder and postpartum psychosis [2]. Although the focus of this paper is on PMADs, which are generally defined as occurring within the first year after birth, the authors acknowledge that maternal depression can last for much longer than the first year and that many of the factors discussed in this paper can affect women for 3 or more years after the birth of a child [3, 4].

The rate of PMADs varies significantly between countries, with some countries experiencing rates as low as 3% (Singapore) and others as high as 38% (Chile) [1]. In general, higher rates of PMADs are observed in countries with higher rates of income inequality, maternal mortality and infant mortality and countries where women of childbearing age work >40 h per week [1]. High fertility rates may also predict higher PMAD prevalence [1]. Other research has shown that among postindustrial countries, the USA has the greatest gap in happiness between parents and nonparents (parents are less happy than nonparents) and that this gap can be largely explained by differences in policies between countries. Countries with more paid time off and child-care subsidies are associated with smaller disparities in happiness between parents and nonparents [5]. It’s possible that the high rates of PMADs in certain countries can be explained by the fact that these countries have already moved far enough away from the hunter-gatherer model (closer birth spacing, longer working hours for mothers and reduced allomaternal support networks) without yet benefitting from the institutional support that has compensated for this loss in many postindustrial countries.

The vast majority of the research on PMADs has been done in postindustrial contexts, so this paper focuses mainly on comparing the social experience of postindustrial motherhood with hunter-gatherer motherhood. There is also greater contrast between the postindustrial maternal experience and those of hunter-gatherers. Future research is needed to examine agricultural and transitional market socioeconomic contexts. It is possible that PMAD incidence in these contexts would be intermediate depending on the degree of mismatch and other risk factors.

The high rate of PMADs around the world is alarming for a number of reasons, not least of which is the risk of suicide. Death from ‘psychiatric causes’ has become a leading cause of maternal mortality in many Western countries [6–9]. A death can be attributed to ‘psychiatric causes’ if it would not have occurred in the absence of a psychiatric disorder [7]. Most deaths attributed to psychiatric causes are suicides, but drug overdose is also a growing source [7]. A study of maternal deaths in the UK between 1997 and 1999 found that psychiatric causes were the leading cause [7]. Another study of 206 perinatal maternal deaths in Alberta, Canada from 1998 to 2015 found that close to one in five maternal deaths was related to suicide or drug toxicity [8]. A 2019 study in CA found similar results: 18% of maternal deaths were due to overdose or suicide [9]. Furthermore, there is evidence that these trends may be increasing. Maternal deaths secondary to drugs and suicide in the USA from 2010 to 2019 were roughly 17%, with a 190% increase in drug-related deaths over the same period [10]. Substance abuse, overdose and suicide are consistently associated with underlying psychiatric disorders such as depression and the postpartum period is a particularly vulnerable time for affective disorder onset [6, 11].

Even when they do not lead to suicide or overdose, PMADs are a source of much suffering for mothers and are frequently associated with adverse psychological and developmental outcomes in children [12]. Especially in developing countries, children of depressed mothers may suffer from undernutrition, stunted development and behavioral problems [13–15]. Children of depressed parents may also be more likely to experience depression themselves in adulthood, thus perpetuating a vicious cycle [16].

Much research to date has focused on the hormonal and biochemical causes of postpartum depression, and less research has focused on the social factors, even though recent reviews suggest that the social factors may be more important determinants [17]. Even when social factors are studied, they are often studied individually rather than looking at how variables may interact [17]. For instance, parenting stress is a known risk factor for postpartum depression (PPD), as is lack of support for new mothers, but few studies have looked at the relationship between short interbirth intervals and lack of support, and how these together might increase parenting stress and lead to higher rates of PPD [17]. Looking at our evolutionary history as a species can help illuminate the abnormality of many contemporary child-rearing practices and guide research on how certain variables interact.

Other research has focused on postpartum depression as a ‘disease of modern civilization’ resulting from a mismatch between contemporary postindustrial lifestyles and our shared evolutionary past [18]. There are many risk factors for PPD which could be explained by evolutionary mismatch. These include dietary mismatches, breastfeeding mismatches, exercise mismatches, sun exposure mismatches and childcare mismatches [18]. These are all important PMAD risk factors, but we believe the social aspects of mismatch merit further examination from an evolutionary perspective. In this paper, we focus specifically on social support for new mothers, the importance of learning to mother and variation in interbirth intervals, both as important individual predictors as well as predictors that could interact in important ways to increase a mother’s risk for PMADs. Such an approach may reveal promising and novel methods of identification, prevention and treatment.

## ARE PERINATAL MOOD DISORDERS ‘MISMATCH DISEASES?’

**Figure 1.**
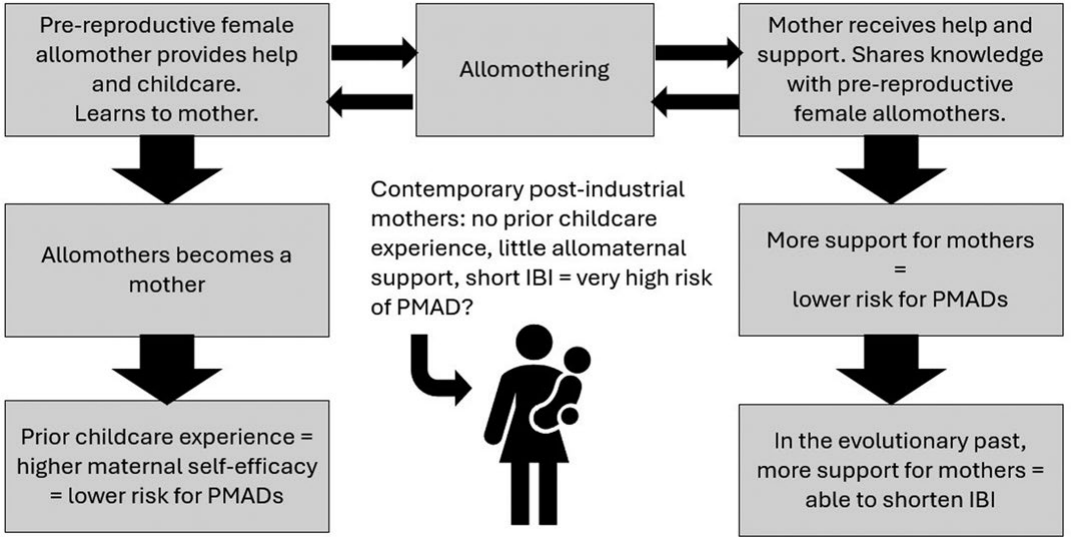
Conceptual framework for how close interbirth spacing, lack of allomaternal support and lack of prior childcare experience contribute to evolutionarily novel high incidence of PMADs

Here we explore the possibility that the maternal mental health crisis can be largely explained by an evolutionary mismatch between the social environment in which human mothers evolved versus contemporary postindustrial society. Beyond the realm of PMADs, it has been hypothesized that differences between the pre-agriculture, pre-industrial context in which most of human evolution took place compared with novel environments brought about by rapid industrialization have contributed to a process of psychological maladaptation accounting for a variety of affective disorders now seen in many postindustrial societies [19]. We cannot study the prevalence of PMADs using the fossil record, and research on the prevalence of these disorders in contemporary hunter-gatherer populations is limited, but certain universal features of the hunter-gatherer lifestyle appear to be protective against a range of affective disorders common in postindustrial societies, and PMADs are no exception [18, 19]. One study of the Hadza, a small-scale, nonindustrialized hunter-gatherer society in Tanzania, found that women report high rates of unhappiness, pain and anxiety leading up to and following childbirth, but the sample size was small and discussion focused on acute, physical pain rather than chronic and emotional states [20]. It is possible that our Paleolithic ancestors experienced PMADs, but perhaps at lower rates than those found in contemporary, postindustrial societies. Better understood is the fact that many common features of hunter-gatherer lifestyles appear to protect against PMADs [18]. Here we focus on the mismatch of the social context of motherhood that we believe merits more in-depth consideration. Specifically, we describe three interrelated areas of mismatch: interbirth intervals, allomaternal support and learning to mother (Fig. 1). ‘Allomother’ refers to any individual other than the mother who participates directly in childcare, including the offspring’s father, other kin and nonkin. Our three domains of interest (interbirth intervals, allomaternal support and learning to mother) are each observed to differ between hunter-gatherer and postindustrial population norms [21–24]. The average interbirth interval is longer in most hunter-gatherer societies than in postindustrial societies, thereby reducing the caregiving burden for mothers with multiple children [21]. In addition, hunter-gatherer mothers have much larger allomaternal networks than most mothers in postindustrial societies, and most hunter-gatherer women accumulate substantial mothering experience prior to their first birth [25]. To further establish this reproductive strategy as ancestral and long-standing among our species, we look to the fossil record. Scholarship in behavioral ecology suggests that a suite of factors co-evolved inter-dependently, but most of these traits are not directly visible in the fossil record: shorter interbirth intervals than our last common ancestor with the *Pan* genus, allomothering and learning to mother. Nonetheless, some traits that coevolved interdependently with those ‘invisible’ traits are discernible in the fossil record, namely (1) earlier age at weaning and (2) prolonged juvenile stage of life compared to what is most likely among our last common ancestor with the *Pan* genus [26, 27].

Comparatively, the experience of many mothers in postindustrial societies is characterized by an adolescent and young-adult life with relatively little allomaternal caregiving experience, followed by sole responsibility for multiple closely spaced offspring and relatively little allomaternal assistance or instruction [18, 23]. We argue that, when taking the long evolutionary view, this kind of motherhood social experience is an evolutionary anomaly, thereby setting the stage for high rates of PMADs.

Indeed, there is some preliminary evidence that each of these factors may play a role in the risk for PMADs, but further research is needed to establish a direct causal link, and to understand how they may interact. We consider all three of these factors to be interrelated since allomothering confers simultaneous benefits both to the mother and the allomother (the former learns to mother while the latter receives a crucial form of help), and allomaternal support is likely what enabled *Homo sapiens* to evolve shorter interbirth intervals relative to our last common ancestor with the *Pan* genus. If human mothers evolved to rely on help from allomothers in order to space births more closely, then it is possible that the combined insult of short interbirth intervals, lack of allomaternal support and inexperience with childcare, as often seen in contemporary postindustrial societies, has a multiplicative effect greater than the sum of its parts.

## INTERBIRTH INTERVALS AND PARENTING STRESS

**Figure 2.**
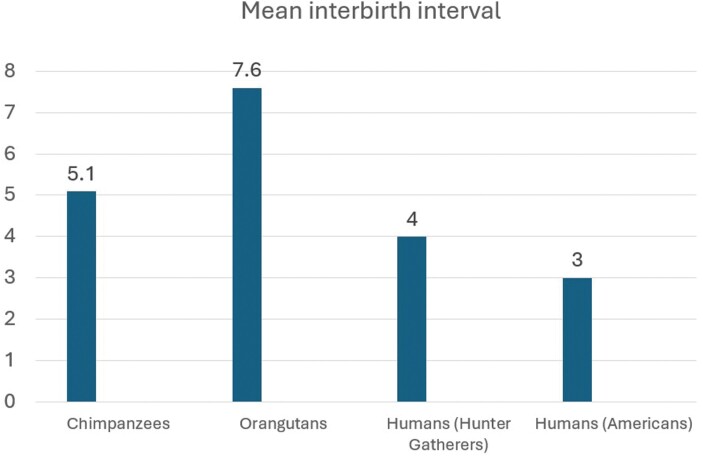
Mean interbirth intervals for females, in years. Sources: chimpanzees [30], orangutans [31], hunter-gatherers [21] Americans [38]

The human interbirth interval is shorter than those of our closest nonhuman Primate relatives, despite the relative altriciality of human infants at birth and longer time to developmental maturity [28, 29] (Fig. 2). The chimpanzee interbirth interval is 5 or 6 years and for orangutans it is 8 [30, 31]. Given how energetically costly it is to raise a human baby to independence, it is surprising that we evolved shorter interbirth intervals relative to a shared common ancestor with other apes [32]. One theory is that humans could not have evolved these shorter interbirth intervals without abundant allomaternal help, since the cost of rearing multiple closely spaced human offspring of different ages with unique age-varying needs is too costly for a mother to manage alone [33]. Others have challenged this model on the grounds that, while more allocare is associated with a reduction in energy expenditure for the mother, it does not appear to affect birth spacing [34]. However, evidence from contemporary hunter-gatherer populations suggests that lots of allomaternal help was likely the norm in our human evolutionary past and it seems plausible that lack of support combined with close interbirth intervals could result in higher rates of PMADs [33, 35–37].

Despite the relative abundance of allomaternal support observed in most contemporary hunter-gatherer societies, the interbirth interval is longer relative to many postindustrial societies [21, 38]. Konner reviewed the research on interbirth spacing among the !Kung, Hadza, Efe, Aka, Ache and Agta and found that interbirth intervals ranged from 48 months (!Kung) down to 36 months (Agta) [21]. At the time the research was done, the populations with lower observed interbirth intervals (Hadza, Efe, Ache and Agta) were experiencing a demographic transition away from pure hunter-gatherer subsistence, which may have even shortened interbirth intervals relative to those communities’ previous patterns [21]. The long interbirth intervals observed in these societies that did not typically practice abstinence or use birth control were likely attributable to frequent and prolonged breastfeeding, combined with nutritional and metabolic stress, which suppresses ovulation [39].

In agricultural and postindustrial societies, mothers typically have shorter interbirth intervals [39, 40]. The phenomenon of closer birth spacing following the transition to agriculture has been well-documented but there is still some debate as to the exact cause. Differences in diet and energy expenditure are one possible explanation, as is the early introduction of animal milk into infant diets, allowing mothers to wean earlier or breastfeed less frequently, thereby resuming ovulation more quickly [41]. Regardless of the mechanism, even the lowest estimates of interbirth spacing in hunter-gatherer societies are generally higher than in agricultural and postindustrial populations. In the USA, for instance, nearly 30% of mothers in 2014 had a second or higher-order birth with an interbirth interval of less than 27 months, and the median interbirth interval was 33–38 months (Fig. 2) [38, 42].

Although there is increasing evidence that spacing births closely increases certain risks (specifically, birth spacing closer than 18 months appears to be associated with a higher risk for premature rupture of membranes and placenta previa), less research is available on the potential mental and emotional impact of spacing births closely [41, 42]. A 2007 study by Mayberry *et al.* of American women indicates that higher parity is positively correlated with depression symptom severity [4]. Another study of Norwegian parents found that closely spaced births were more often associated with mothers’ mortality and medication use in mid-life or early old age [43]. Finally, there is ample research to show that high levels of parenting stress and childcare stress, defined as the imbalance between the demands of caring for young children and available resources, are risk factors for postpartum depression [17]. It is probable that close birth spacing contributes to maternal overwhelm by increasing the caregiving burden for the mother, especially in the absence of adequate alloparental support, and this could lead to an increased risk for PMADs, but more research is needed to establish a direct link.

## ALLOMOTHERING AND SOCIAL SUPPORT

Absence of social support, especially from maternal kin, has been consistently associated with risk for PMADs [17, 18]. There is abundant evidence to suggest that we evolved to cooperatively rear our offspring, yet lack of social support is a common feature of postindustrial motherhood [18, 44]. We acknowledge that humans do not exactly fit the criteria for either cooperative or communal breeders, although if we expand the traditional definition of cooperative breeding to mean social systems in which nonbreeding allomothers help the male–female breeding pair in raising offspring, humans would certainly seem to fit the classification [35]. Other scholars have suggested that the human social reproductive system is unique and merits its own terminology. Bogin has suggested ‘biocultural reproduction’ to describe the flexibility of allocare in small-scale human societies [45].

Studies of hunter-gatherer societies have repeatedly shown that caring for babies is a collaborative effort. A study of !Kung mothers and infants showed that for nearly half of the recorded incidences of infant crying, someone other than the mother responded, either together with the mother or alone and in one-third of instances the mother was not present [33]. Similarly, studies of Hadza hunter-gatherers report that newborns were held by allomothers 85% of the time in the first days after birth [29]. When looking at daytime minutes in the baby’s first year of life, Hadza mothers were involved in 78% of minutes, fathers and older sisters in roughly 18% each, grandmothers and older brothers in roughly 9% and all others in 29–41% [22]. The Efe is thought to be one of the most extreme examples of high allomaternal care among small-scale societies, with allomothers accounting for 39% of infants’ physical contact at 3 weeks and 60% at 8 weeks, and nonmaternal nursing is also frequently observed [36]. Studies of the Efe found that babies average 14 different caretakers in the first days of life [29]. Shared suckling has been observed in at least 87% of typical foraging societies documented in the Human Relations Area Files [29]. In the Aka, while in camp, 1–4-month-olds are held by their mothers less than 40% of the time, are transferred to other caregivers an average of 7.3 times per hour, and have seven different caregivers on average that hold the infant during the day [22].

Although unrelated allomothers are important sources of help in most hunter-gatherer societies, kin account for a larger portion of care and may also offer higher quality care [35]. A comparative study of the Agta and BaYaka found that in both societies, ~70% of care was provided by kin [35]. Furthermore, kin are more likely to provide emotional or educational support in addition to substitutive childcare [46]. Across many small-scale societies, grandmother allomaternal support is highly sought-after, as it typically confers emotional and educational benefits in addition to direct substitutive benefits [37, 46]. Many studies have shown the presence of a maternal grandmother can significantly improve child survival rates, more so than other kinds of allomothers [47].

Children are also an important source of allomaternal care in most hunter-gatherer societies. Indeed, in some hunter-gatherer societies, the care of younger children by older children may be one of the most important forms of substitutive childcare for mothers [37]. Because the care of children by children occurs within the context of a multi-age playgroup, the burden to any one individual child is relatively low and is typically compatible with their own play [37].

**Figure 3.**
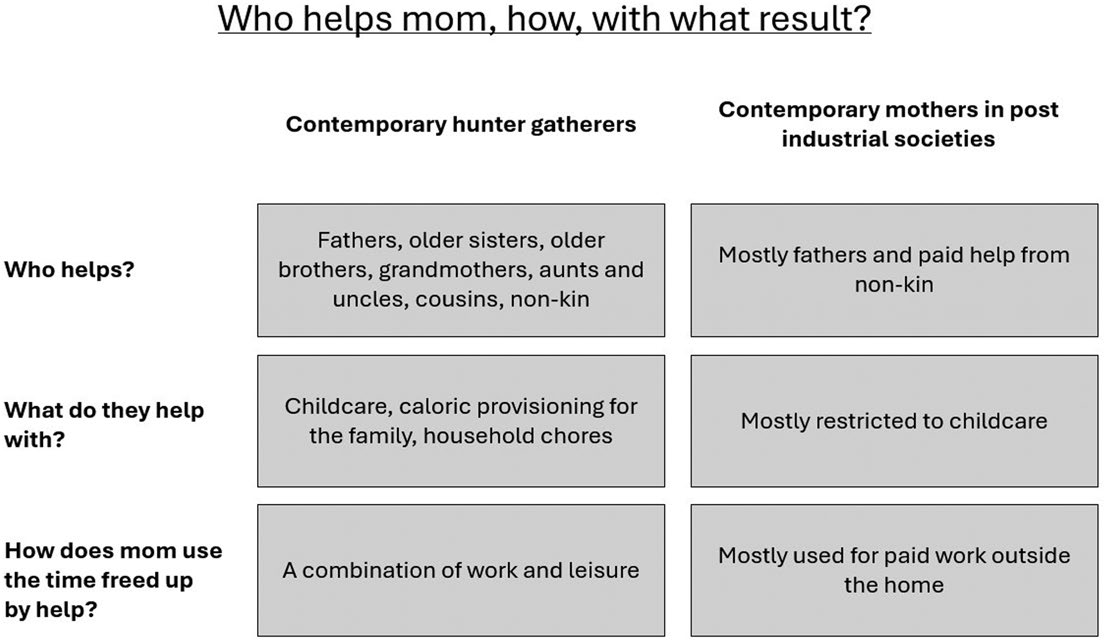
Comparison of the roles of allomothers in hunter-gatherer and postindustrial societies

Compared with hunter-gatherer societies, mothers in postindustrial societies often raise their closely spaced children in relative social isolation, often with little help from kin (Fig. 3). A comparative study of allomaternal care of infants in !Kung hunter-gatherers and 10-month-old girls in Boston found that !Kung mothers accounted for 75-80% of physical contact with infants in the first 20 months, but 20–25% of physical contact came from caregivers other than the mother, much higher than was true for the Boston infants [21, 22, 48]. This is true in toddlerhood as well: by the time children are 2 years old, face time with the mother is significantly less in hunter-gatherer societies than in postindustrial societies. A study comparing !Kung 2–5-year-olds with London 2–5-year-olds showed that !Kung children ranged a greater maximum distance from mothers and were nurtured less by their mothers (or anyone) than the London children [22] Mothers in postindustrial societies therefore face a higher caregiving burden than their hunter-gatherer counterparts, largely due to lack of allomaternal support.

In some ways, institutions compensate for the loss of allomaternal participation in childcare or may even partially cause it. Schools, daycares, nannies, babysitters, pediatricians and grocery stores provide some of the duties that would otherwise be provided by allomothers in small-scale societies. However, for the first 5 years of life in the USA (and for similar periods of time elsewhere across postindustrial societies) childcare remains largely the responsibility of mothers due to the gap in state-sponsored childcare from birth to elementary school [49]. During this gap, some families may pay for private childcare or assistance in the form of daycare or nannies, but due to the substantial financial burden of these options, it is not clear that the psychological burden to mothers is alleviated [50]. Additionally, it remains unknown what the psychological impact on mothers may be of the transfer of childcare assistance from allomothers (kin and other trusted community members) to institutions. Large, institutional daycares with high ratios of children to caregivers and high rates of staff turnover are not conducive to babies and children forming secure attachments to alloparents [51]. Finally, for employed mothers, hours of nonparental childcare in postindustrial societies are mainly used for paid work, as opposed to rest or leisure [52]. Although mothers in hunter-gatherer societies also rely on shared care in order to work, in many foraging societies mothers have far more leisure time than did mothers after the transition to agriculture and industrialization. Leisure time is often spent in close proximity to children, but owing to the communal nature of living and the abundance of playmates, mothers are not expected to be their children’s constant entertainers. A study of Agta hunter-gatherers found that mothers spent 35% of daylight hours in leisure, which included rest, sleep, adult socialization and play, regardless of the age of their babies or children [53, 54]. In postindustrial societies, mothers spend far less time in leisure [52]. Although it’s hard to compare precisely owing to different methodologies, we can estimate that 35% of daylight hours would amount to about 5.25 h of leisure for Agta mothers, whereas research on mothers in contemporary postindustrial societies estimates they spend an average of 3–4 h per day in leisure [55].

It is possible that lack of social interaction during the postpartum period, more than lack of help, may put mothers in postindustrial societies at higher risk for PMADs. It has been well-established that social isolation and loneliness are known risk factors for all types of depression, and mothers are no exception [56]. Indeed, loneliness in pregnant mothers and mothers of children under 5 years in postindustrial societies is estimated to be as high as 40%, and as high as 70% if mothers have children with a problem [24]. In countries with little federal funding for childcare, women are often forced to exit the workforce after having children, which can increase feelings of loneliness and isolation [57]. Social support from other women with children or from friends does not appear to buffer against PMADs the same way that support from maternal kin does [17]. Even when kin live far away, evidence suggests that psychological and emotional support are negatively associated with postpartum depression [58]. These findings suggest that support, whether instrumental or emotional, more than socialization, is the crucial differentiator.

## LEARNING TO MOTHER

**Figure 4.**
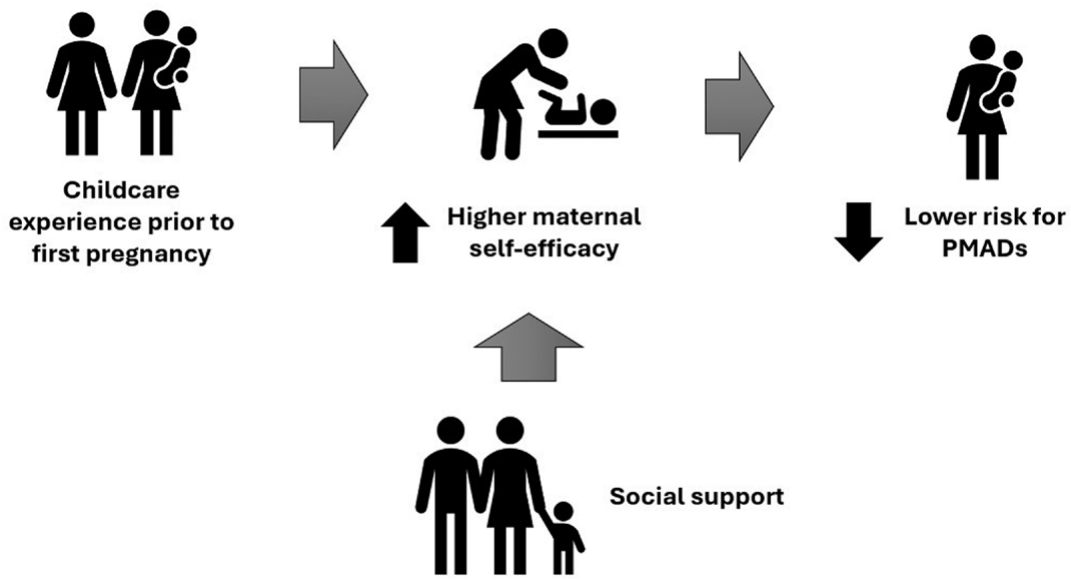
How learning to mother affects PMAD risk

In addition to providing much-needed support to mothers, the practice of allomothering confers another important benefit: it gives prereproductive females the practice of caring for infants prior to their first birth [25]. This experience may in turn increase a mother’s sense of self-efficacy and give her a more realistic idea of the challenges of motherhood, both of which are known to buffer against perinatal mood disorders [59–61] (Fig. 4).

Research suggests that many aspects of mothering are ‘learned’ skills in primates. In free-ranging vervet monkeys, juvenile females show a high degree of interest in caring for infants and, by the time they are adolescents this practice has made them into competent allomothers [62]. By contrast, primates raised in captivity, deprived of social learning opportunities, are incompetent mothers and may go so far as to completely reject their offspring [62]. Other evidence from captive groups of vervet monkeys suggests that allomothering as a juvenile makes first-time mothers more likely to successfully rear their infant [63].

Given the importance of allomothering in nonhuman primates and the relative complexity of human mothering, it is likely that humans also benefit from learning to mother through allomaternal caregiving. The most present and helpful allomothers in many small-scale human societies are prereproductive girls [25, 35]. A comparative study of the BaYaka and Agta societies found that subadults accounted for roughly 54% of care in both societies [35]. This finding is fairly consistent across other small-scale societies studied and may even be higher in some [35]. Though the former study does not identify gender, other studies have shown that these subadult helpers are disproportionately female. A study of the Hadza in Tanzania found that subadults represented 62% of total allomothers, of which 44 were female and 12 were male [25]. Moreover, the female allomothers spent a significantly higher proportion of their time holding infants than did their male counterparts, and holding by girls was greatest between the ages of 8 and 12 [25].

By contrast, mothers in postindustrial societies spend relatively little time allomothering before their first birth (Fig. 4). Postindustrial demographic shifts to life in nuclear families and dispersal of kin networks have made mothers more reliant on paid help and help from partners [23]. At the same time, delayed onset of first pregnancy, close interbirth intervals and norms around school participation have made childcare by children and adolescents increasingly rare [23]. As such, there are relatively few opportunities for sub-adult females to practice their mothering skills prior to first birth. A US nationally representative sample of households with children found that 22% of these families had adolescents (age 12–18) along with younger children (under age 12), and among those, adolescents did not regularly participate in childcare in 80% of such families [29], suggestive of the rarity of adolescent participation in childcare in the US cultural context. However, when the Covid-19 pandemic forced families into sheltering in place, data show that for families in the UK with older siblings at home, mothers got more help from these older siblings than from fathers, grandparents, or any other category of allomother [64].

Although we were not able to identify any studies directly linking allomothering experience with PMADs, there is substantial evidence suggesting that maternal self-efficacy is inversely correlated with PMADs, especially for primiparous mothers [59, 60]. Parenting self-efficacy can be broadly defined as the extent to which a parent feels confident in dealing with parenting issues [65]. It seems probable that parenting experience prior to first birth could increase a mother’s sense of self-efficacy. Furthermore, self-efficacy is moderated by social support, especially from kin but also sometimes from partners and professionals, presumably because teaching from more experienced individuals can increase a mother’s confidence and competence [59]. Finally, allomothering experience prior to first birth may help temper mothers’ expectations, thereby reducing the risk of PPD. Research has shown that the discrepancy between a mother’s expectations and the reality of caring for a newborn can be a major factor in her experience of PPD [61]. More research is needed to establish whether childcare experience prior to first birth buffers against PMADs.

## CONCLUSIONS AND IMPLICATIONS

High rates of PMADs in postindustrial societies have generated new interest in factors associated with risk for perinatal mood disorders. Increasingly, research suggests that postpartum depression and other perinatal mood disorders may be ‘diseases of modernity’ arising from a mismatch between the environments in which humans and our ancestors evolved over hundreds of thousands of years and postindustrial environments. Evolution by natural selection operates on differences in reproductive success, so it is logical that behaviors and traits related to women’s perinatal mental health would be strongly conserved, making them good candidates for mismatch. Indeed, it is now widely accepted that cooperative breeding and extensive allomaternal help is what enabled humans to evolve shorter interbirth intervals relative to our last common ancestor with *Pan*, making allomothering an adaptive behavior [44]. The arrival of agriculture and industrialization made it possible for mothers to space their births even more closely without compromising neonatal survivorship, while concomitantly disrupting traditional maternal support networks [66]. We believe this rapid cultural divergence from a maternal experience that evolved over hundreds of thousands of years has set the stage for higher rates of PMADs. Many mothers in postindustrial societies now raise their closely spaced offspring in relative social isolation with little prior mothering experience: a truly abnormal experience from an evolutionary perspective [21–24, 48]. Does close interbirth spacing, combined with low levels of kin support and lack of mothering experience put mothers at particular risk for PMADs? Further research is needed to understand how these factors interact and exactly how they are associated with PMAD frequency and severity. Such research could have important implications for public health and medical advice, by helping medical practitioners screen for PMAD risk factors, or through the implementation of preventative programs aimed at supporting mothers at particular risk.
